# Charge Reduction and Thermodynamic Stabilization of Substrate RNAs Inhibit RNA Editing

**DOI:** 10.1371/journal.pone.0118940

**Published:** 2015-03-05

**Authors:** W.-Matthias Leeder, Andreas J. Reuss, Michael Brecht, Katja Kratz, Josef Wachtveitl, H. Ulrich Göringer

**Affiliations:** 1 Molecular Genetics, Darmstadt University of Technology, Darmstadt, Germany; 2 Institute of Physical and Theoretical Chemistry, Goethe University Frankfurt am Main, Frankfurt am Main, Germany; NIH, UNITED STATES

## Abstract

African trypanosomes cause a parasitic disease known as sleeping sickness. Mitochondrial transcript maturation in these organisms requires a RNA editing reaction that is characterized by the insertion and deletion of U-nucleotides into otherwise non-functional mRNAs. Editing represents an ideal target for a parasite-specific therapeutic intervention since the reaction cycle is absent in the infected host. In addition, editing relies on a macromolecular protein complex, the editosome, that only exists in the parasite. Therefore, all attempts to search for editing interfering compounds have been focused on molecules that bind to proteins of the editing machinery. However, in analogy to other RNA-driven biochemical pathways it should be possible to stall the reaction by targeting its substrate RNAs. Here we demonstrate inhibition of editing by specific aminoglycosides. The molecules bind into the major groove of the gRNA/pre-mRNA editing substrates thereby causing a stabilization of the RNA molecules through charge compensation and an increase in stacking. The data shed light on mechanistic details of the editing process and identify critical parameters for the development of new trypanocidal compounds.

## Introduction

Infections with protozoal pathogens have a global impact, which in part is reflected in the long-standing search for antiprotozoal compounds. Unfortunately, effective treatments for the different diseases are by and large not available [1]. This holds especially true for African trypanosomiasis a parasite infection also known as African sleeping sickness. The disease is a medical problem in many parts of sub-Saharan Africa due to the lack of effective therapeutics and an increasing resistance of the parasite to long-established chemotherapeutics [2]. Causative agent of sleeping sickness is the organism *Trypanosoma brucei*—an extracellular parasite that multiplies in the blood and the lymphatic and cerebrospinal fluids. Some progress in the development of new trypanocidal compounds has recently been made by reformulating existing drugs while at least two other compounds are currently being tested in clinical trials [3]. In addition, alternative approaches to address the need for safe and effective drugs have been explored for instance by using conjugated parasite-specific nanobodies [4] or SELEX-derived, trypanosome-specific RNA aptamers [5–7].

Despite these efforts, almost no attention has been paid to RNA-mediated biochemical pathways as potential drug targets. Parasite-specific RNAs and/or ribonucleoprotein (RNP) complexes likely represent vulnerable targets that can be exploited with RNA-interacting, small molecule compounds [8–10]. Within that context the RNA editing reaction within the mitochondria of trypanososmes is of special importance. The processing reaction is a key pathway in the life cycle of the parasite [11] and the process is catalyzed by a unique high molecular mass protein complex, the 20S editosome [12]. Moreover, editing involves a class of small, non-coding RNAs, known as guide (g)RNAs, which only exist in the parasite. An editing reaction cycle is characterized by the site-specific insertion and to a lesser degree deletion of exclusively U-nucleotides into otherwise non-translatable mRNAs. The reaction is catalyzed within a single reaction center on the surface of the 20S “protein-only” editosome [13]. RNA substrates are pre-edited mRNAs and guide RNAs, which provide the specificity for the U-insertion/deletion reaction.

Although editing is most prominent during the insect life cycle stage of the parasite, it also occurs in the infective bloodstream stage and thus can be considered an ideal drug target [14]. However, only a limited number of inhibiting compounds have been identified to date [15]. This includes inorganic pyrophosphate (pp_i_), which is generated from UTP during the U-insertion reaction and as a consequence inhibits the terminal uirdylyltransferase (TUTase) of the editosome by feedback inhibition. Chemically inert cosolutes such as polyethylenglycol (PEG) have recently been shown to block the reaction indicating that *in vitro* RNA editing is sensitive to molecular crowding conditions [16]. Other inhibitors have been identified by a combined virtual screening/molecular dynamic simulation approach [17] or by high throughput screening of chemical libraries. This identified compounds such as mitoxanthrone, protoporphyrin IX and D-sphingosine, which inhibit with half-maximal inhibitory concentrations (IC_50_) in the low micromolar range and likely function by binding to the editosome or individual proteins of the catalytic complex [18]. Similarly, several naphthalene-derivatives have been identified as low micromolar inhibitors of the editing core enzyme REL1 (RNA editing ligase 1). They interfere with the deadenylation of the enzyme in addition to blocking the integrity and/or assembly of the editosome [19–21].

Although, the 0.8MDa editosome presents a large drug-binding landscape, conceptually it should also be possible to modulate the processing reaction by RNA substrate-binding. Typical examples for RNA-binding, small molecule inhibitors are some antibiotics such as the aminoglycosides, which interfere with protein biosynthesis by directly binding to the ribosomal decoding site of the small subunit ribosomal RNA [22]. Although aminoglycosides show selectivity in their binding of RNA over DNA, they are rather nonselective towards different RNA molecules. Aminoglycosides have been identified to interact and inhibit a wide range of unrelated RNAs and biochemical processes including protein synthesis, group I intron splicing, RNA catalysis as well as viral RNAs [10]. This promiscuity is the result of a general electrostatic binding modality and a conformational adaptability of the aminosugars [23], which bind into the major groove of A-form RNA duplex elements as well as major grooves that are widened by the proximity of a loop or a bulge [24–26].

Here we show that both, U-insertion- and U-deletion editing can be inhibited by specific aminoglycosides with IC_50_-values in the low micromolar range. The inhibition is a consequence of high affinity binding of multiple aminoglycoside molecules into the major groove of the gRNA/pre-mRNA substrate RNAs, which results in a thermodynamic stabilization of the RNAs. The data shed light on mechanistic details of the editing reaction cycle and identify critical parameters for the development of new trypanocidal compounds.

## Materials and Methods

### RNA synthesis

Short, synthetic pre-mRNAs and gRNAs were synthesized by solid phase phosphoramidite chemistry using 2′-O-triisopropylsilyloxymethyl (TOM)-protected monomers. The primary sequences of the different RNAs are listed in S1 Table. RNA oligonucleotides were radioactively phosphorylated (^32^P) following standard procedures. Pre-mRNA/gRNA hybrid RNAs were formed by annealing equimolar amounts of RNA oligonucleotides in editing buffer (EB): 20mM HEPES/KOH, pH7.5, 30mM KCl, 10mM Mg(OAc)_2_, 0.5mM DTT at 65°C for 5min and cooling to RT at a rate of 1°C/min.

### Inhibition of RNA editing *in vitro*

RNA editing *in vitro* insertion and deletion assays including the preparation of 20S editosomes were performed as described [27,28]. Pre-annealed, synthetic pre-mRNA/gRNA hybrid RNAs were incubated with 20S editosomes in EB in the presence of varying concentrations of different antibiotics. Editing was allowed to proceed for 3 hours at 27°C and was terminated by the addition of H_2_O-saturated phenol. RNAs were EtOH precipitated and analyzed in denaturing, 15% (w/v) polyacrylamide gels followed by phosphorimaging. Band intensities were densitometrically quantified and used to plot dose response curves. The data were fitted to a modified Hill equation using IGOR Pro 6.32A (WaveMetrics) to derive IC_50_-values. The following aminoglycosides were used: 4,5-linked 2-DOS (neomycin B, ribostamycin, paromomycin, lividomycin); 4,6-linked 2-DOS (kanamycin A and B, sisomycin, tobramycin, G418); aminocyclitols (apramycin, streptomycin, hygromycin B); macrolides (erythromycin B, azithromycin and spiramycin) and neamin. The tested concentration range was >3 orders of magnitude from submicromolar to millimolar.

### Isothermal calorimetry

Isothermal calorimetric measurements were performed at 27°C in a MicroCal ITC200 instrument (GE Healthcare Life Sciences). Guide RNA/pre-mRNA substrate RNAs were dissolved in 0.2mL EB at a concentration of 2μM. Following thermal equilibration, a 0.13mM neomycin B stock solution in EB was titrated into the RNA solution in 2.5μL aliquots at 3min intervals while stirring. Raw data were recorded as power (heat flow) in μcal/s over time/min. The area under each heat burst peak was integrated and plotted against the molar neomycin B/RNA ratio. All binding isotherms were corrected for the effect of titrating neomycin B into buffer alone. Changes in enthalpy (ΔH) and the macroscopic equilibrium dissociation constant (K_d_) were extracted by curve fitting (Origin v5.0). Gibbs free energies (ΔG) and entropy changes (ΔS) were calculated from ΔG = -RTxlnK and ΔG = ΔH-TΔS (T = temperature in degree Kelvin; R = universal gas constant (1.986 cal/K/mol)). Macroscopic K_d_-values were converted to intrinsic binding constants (K_i_) following a sequential binding site model as K_d_ = (n-i+1/i)xK_i_ [29,30].

### UV melting

Absorbance *versus* temperature profiles of gRNA/pre-mRNA hybrid RNAs were recorded at 260nm using a thermoelectrically controlled UV-spectrophotometer. The temperature was scanned at a heating rate of 0.75°C/min at temperatures between 15°C and 95°C. Absorbance values were recorded with an average time of 0.5s and data were collected every 0.1°C. Samples contained 1μM RNA in 5mM Na cacodylate, pH6.8. Na^+^-ion titration experiments were performed at 5–250mM NaCl and neomycin B titrations were conducted at a concentration range of 1.3μM-13μM. Melting temperatures (T_m_) and all thermodynamic parameters (ΔH, ΔS, ΔG) were calculated as in [16]. The number of RNA/neomycin B ion contacts was calculated as in [31,32]. The number of ions (Δn) released or taken up in the melting process were calculated using ∂T_m_/∂log[Na^+^] = -0.9x(2,303RT_m_^2^/ΔH^0^)Δn [33].

### Circular dichroism

CD measurements of pre-annealed gRNA/pre-mRNA hybrid RNAs were carried out at 27°C in 10mM Na cacodylate, pH6.8 and 65mM NaCl. Spectra were recorded from 205–315nm at an RNA concentration of 12μM. Neomycin B was added from a concentrated stock solution in 10mM Na cacodylate, pH6.8 and 75mM NaCl to yield final concentrations between 0–0.6mM. Samples were equilibrated for 5min before measuring. Resulting spectra were Savitzky-Golay smoothed [34] and corrected for dilution. Changes in the ellipticity (ΔƐ) at 273nm were plotted as a function of the neomycin B/RNA ratio to derive the number of aminosugar binding sites.

### RNA modeling/docking

Guide RNA/pre-mRNA hybrid RNAs were interactively modeled using ERNA3D^©^ 2.0 (Pentafolium-Soft). Neomycin B molecules were docked into the modeled RNAs using Autodock Vina 1.1.2 [35]. File conversion from pdb-files to pdbpt-files was performed using Autodock Tools 1.5.6 rc3 [36]. All docking experiments were performed as blind dockings choosing a search space that encapsulated the individual helices of the two RNAs. Four individual runs were performed using the Autodock Vina default parameters with an exaustiveness value of 15. All rotatable bonds of neomycin B were allowed to rotate freely while the RNA was considered rigid. Images were created using Pymol 1.3 (www.pymol.org). Autodock Tools [36] was used in conjunction with pdb2pqr [37] for generating the input files needed by APBS 1.3 [38]. Surface potentials were calculated using the default settings of the PyMOL APBS plug-in.

## Results

### Aminoglycosides inhibit *in vitro* RNA editing

**Fig 1:**
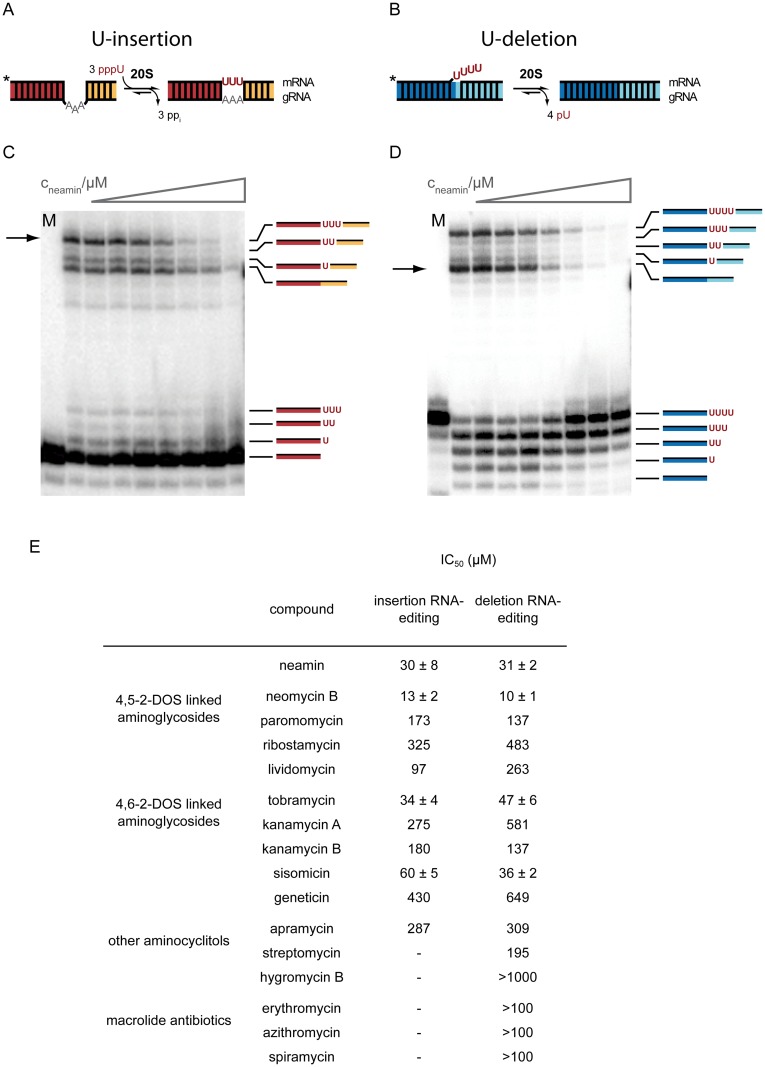
Trypanosome-specific U-insertion and U-deletion RNA editing. Depicted are two gRNA/pre-mRNA pairs emphasizing the helical domains of the U-insertion RNA substrate in red and yellow (A) and the helices of the U-deletion RNA in dark and light blue (B). *In vitro* U-insertion monitors the insertion of 3 U’s; *in vitro* U-deletion the removal of 4 U nt. The reaction is catalyzed by the 20S editosome. Primary sequences of the individual RNAs are given in S2 Fig. Inhibition of U-insertion (C) and U-deletion RNA editing (D) by neamin. Radioactively labelled (5’-^32^P) gRNA/pre-mRNA substrate RNAs were incubated with 20S editosomes in the presence of increasing concentrations of neamin (1.6μM-1.7mM, left to right). RNA reactants, intermediates and edited products (annotated to the right of the two gels) were electrophoretically separated and densitometrically quantified to yield dose response curves (S3 Fig) from which IC_50_-values were derived (E). Errors are standard deviations (s.d.). M: mock treated sample. *: position of the radioactive label (^32^P). Arrows indicate the position of the fully edited mRNA products.

Based on the published data on the inhibition of translation, pre-mRNA splicing and other RNA-driven reactions [10] we tested the ability of a panel of antibiotics including several aminoglycosides to interfere with U-insertion/deletion-type RNA editing. Together we analyzed four 4,5-linked 2-deoxystreptamines (2-DOS), five 4,6-linked 2-deoxystreptamines, three aminocyclitols and three macrolides (for chemical structures see S1 Fig). The two different editing reactions (U-insertion/U-deletion) were tested independently using “precleaved” *in vitro* RNA editing assays [27,28]. Both assays rely on short, synthetic pre-mRNA/gRNA hybrid RNAs, which are converted into edited products either by the insertion of 3 U-nucleotides (nt) or the deletion of 4 U’s (Fig. 1A/B). The reaction is catalyzed by 20S editosomes and was performed at varying antibiotic concentrations. RNA reactants, intermediates, edited products as well as non-productive side products of the reaction were electrophoretically separated and densitometrically quantified. Fig. 1C/D show two representative experiments using the aminoglycoside neamin. Neamin inhibits both editing reactions in a dose-dependent manner (S3 Fig). The U-insertion reaction is half-maximally (IC_50_) inhibited at a concentration of 30±8μM and the IC_50_-value for inhibiting U-deletion editing is 31±2μM. All data are summarized in Fig. 1E. Neomycin B (neo) is the most effective inhibitor with IC_50_-values of 10±1μM and 13±2μM (U-deletion/U-insertion) followed by neamin (31/30μM), tobramycin (47/34μM) and sisomycin (36/60μM). The macrolides erythromycin, azithromycin and spiramycin and the aminocyclitols apramycin, streptomycin and hygromycin B showed no effect (IC_50_≥100μM).

Importantly, the inhibitory effect shows pleiotropic characteristics: next to inhibiting the formation of the fully edited pre-mRNA other steps of the processing cycle are affected as well. In the case of the U-deletion, the exoUase trimming reaction at the 3’-end of the 5’-pre-mRNA fragment is inhibited, although to a lesser degree. Furthermore, religation of the “faithfully” edited reaction product is more sensitive to the aminosugars in comparison to the formation of the unedited ligation side-product. However, while the formation of edited products in both cases is completely stalled at concentrations ≥100μM, the inhibition of the exoUase is not. Lastly, we also analyzed the endonuclease cleavage step of the editing cycle. mRNA cleavage is similarly inhibited in a dose dependent manner and is completely abolished at ≥30μM neomycin B (S4 Fig).

### Aminoglycosides bind to editing substrate RNAs

**Fig 2:**
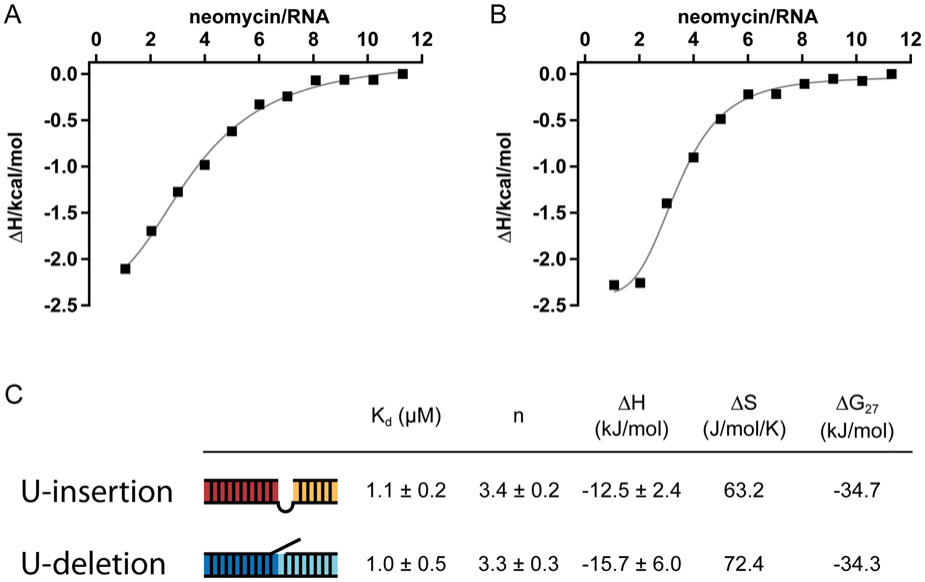
ITC-titration profiles of U-insertion (A) and U-deletion (B) gRNA/pre-mRNA hybrid RNAs with neomycin B. (C) Summary of the derived thermodynamic characteristics of the binding reaction: equilibrium dissociation constant (K_d_), number of binding sites (n), enthalpy (ΔH), entropy (ΔS) and Gibbs free energy (ΔG). Errors are standard deviations (s.d.).

Aminoglycosides have been shown to execute their inhibitory effects through a promiscuous RNA binding activity [23]. Therefore, we analyzed the possible binding of neomycin B (the most potent inhibitor of the editing reaction) to the two editing substrate RNAs using isothermal calorimetry (ITC). As shown in Fig. 2A/B neomycin B binds to both, the U-insertion and U-deletion gRNA/pre-mRNA hybrid RNAs with micromolar affinity. The binding is characterized by a macroscopic equilibrium dissociation constants (K_d_) of 1.1μM for the U-insertion gRNA/pre-mRNA hybrid and of 1.0μM for the U-deletion RNA (Fig. 2C). The binding reaction is exothermic and at saturation 3 RNA binding sites are occupied with neomycin B. The interaction is enthalpically driven [25] with Gibbs free energies in the range of-35kJ/mol. Assuming that the 3 binding sites are identical, the determined macroscopic K_d_’s were redefined relative to the progress of saturation using a sequential saturation binding site model [29,30]. This allowed the calculation of intrinsic binding constants (K_i_) from which the fraction of RNA molecules having 1, 2 or 3 binding sites occupied was determined (S5 Fig). At a neomycin B concentration of app. 12μM half maximal occupancy of all 3 binding sites is achieved in aggreement with the above determined IC_50_-value.

Importantly, neomycin B has no affinity for 20S editosomes as demonstrated by surface plasmon resonance (SPR) binding experiments. At neomycin B concentrations up to 10-fold above the determined K_d_ of the RNA/neomycin B complex no binding was observed. Only at a ≥100-fold excess a weak interaction was identified (S6 Fig). Similarly, neomycin B does not affect the binding of substrate RNA to 20S editosomes. Even at a neo concentration 250xK_d_ no dissociation of bound RNA from the complex can be detected (S7 Fig).

### Aminoglycosides stabilize editing substrate RNAs

**Fig 3:**
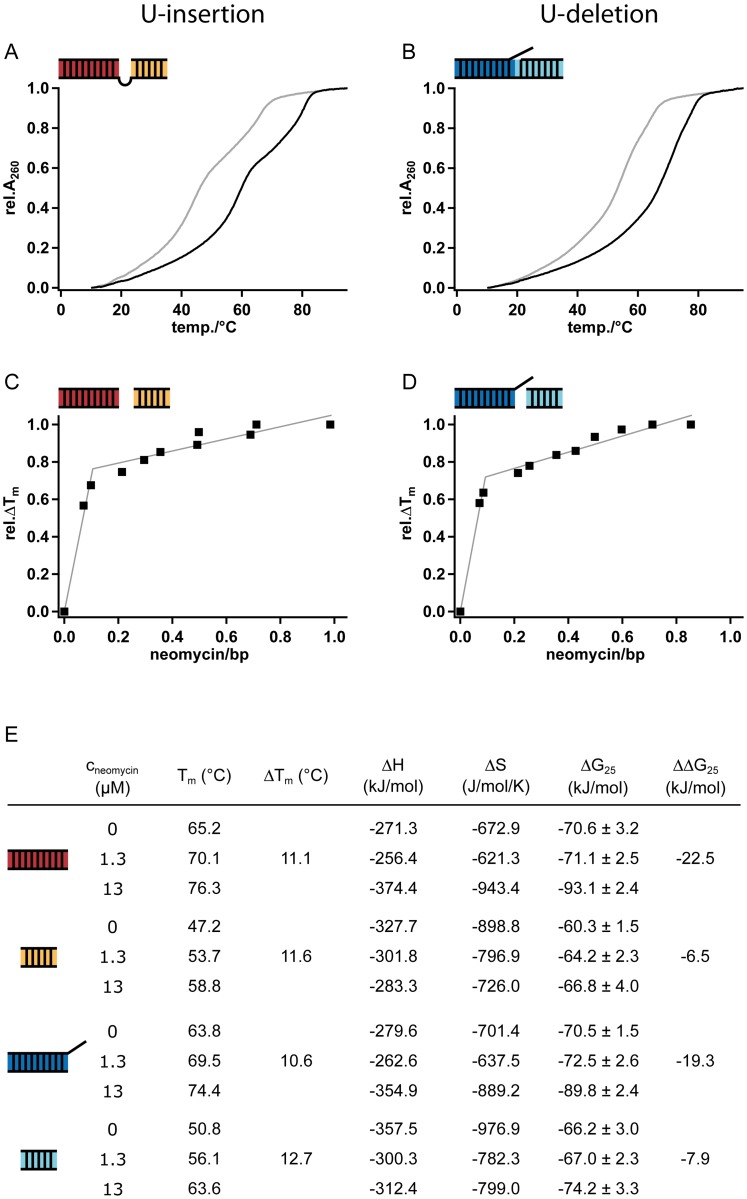
UV-melting profiles of U-insertion (A) and U-deletion (B) gRNA/pre-mRNA substrate RNAs (1μM) in the absence (grey trace) and presence (black trace) of neomycin B. Both RNAs exhibit two distinct helix/coil transitions indicating the independent melting of the two helical domains in both RNAs. In the presence of neomycin B (13μM; 13-fold over K_d_) both melting curves shift to higher temperatures indicating a stabilization of the editing RNAs. (C)/(D) Plotting the T_m_-value changes of the individual helices as a function of the molar neomycin B/bp ratio results in 3.1 (U-insertion) and 3.3 (U-deletion) neomycin binding sites per editing RNA. (E) Summery of the measured/calculated T_m_, ΔT_m_ and thermodynamic values for the individual helices in both gRNA/pre-mRNA substrate RNAs.

Based on the observation that the aminoglycoside-induced inhibition of editing might be a result of the high affinity interaction of the drug to the substrate RNAs we decided to analyze the consequence(s) of the RNA/aminosugar interaction. For that we performed UV-melting experiments of gRNA/pre-mRNAs hybrid RNAs in the presence of defined concentrations of neomycin B. Representative melting curves for the U-insertion and U-deletion RNAs are shown in Fig. 3A/B. In the absence of neomycin B, both RNAs show two separate helix/coil transitions indicating independent melting events of the two helical domains in both RNAs. T_m_-values were derived from 1^st^-derivative plots and identified melting midpoints at temperatures ranging from 47°C to 65°C. In the presence of 1.3μM neomycin B (1.3-fold above K_d_) the individual melting transitions shift to higher temperatures with ΔTm’s between 11–13°C. This indicates a strong stabilization of the helical elements in both RNAs equivalent to ΔΔG-values of-7 to-23kJ/mol. All binding reactions show saturation characteristics, which allowed the determination of the number of neomycin B binding sites per RNA (Fig. 3C/D). For the U-insertion RNA we identified 3.3 binding sites and for the U-deletion substrate 3.1 binding sites in agreement with the above-described ITC-measurements. All data are summarized in Fig. 3E.

### The neomycin B/editing substrate RNA interaction relies on ionic contacts

**Fig 4:**
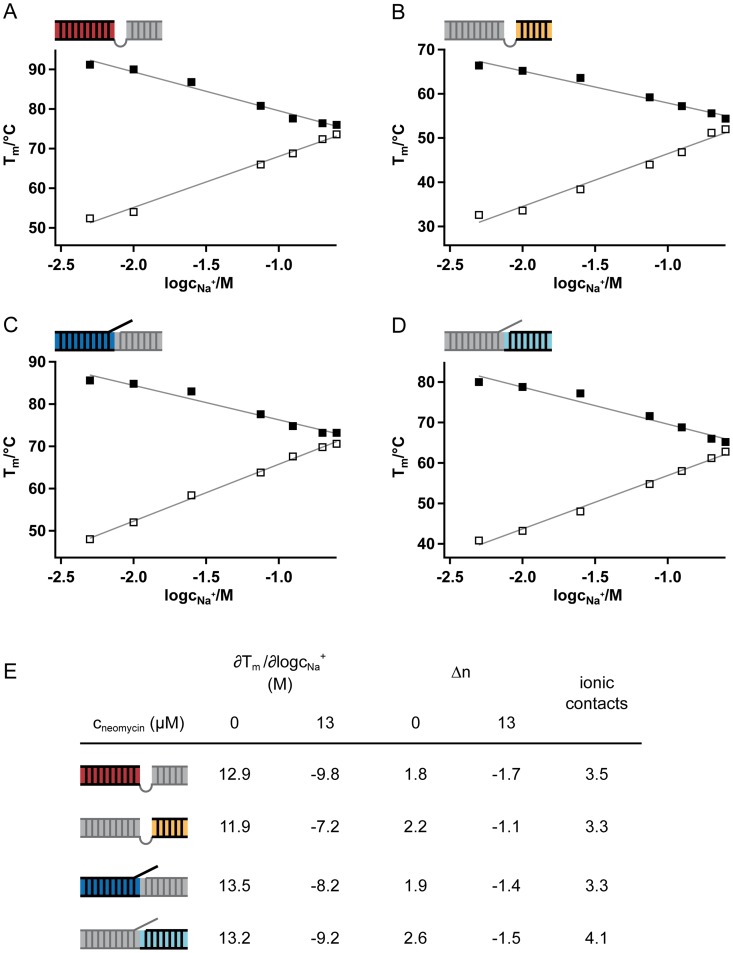
Salt dependence of UV-melting profiles. Sodium ion dependence (10–250mM) of melting temperatures of the U-insertion (A/B) and U-deletion (C/D) gRNA/pre-mRNA editing substrate RNAs (1μM) in the absence (open squares) and presence (filled squares) of neomycin B (13μM). Plots of the melting temperature (T_m_) *versus* log of the Na^+^-ion concentration (log c_Na+_) for the 4 individual helices of the two editing RNAs. Solid lines: linear regressions of the data points. (E) Summary of the derived data.

The ability of aminoglycosides to bind to RNA molecules has been attributed to two general phenomena: (i) the conformational adaptability of the sugar molecules and (ii) their “overall” electrostatically-driven RNA binding mode [10,23]. In order to test the contribution of a charge/charge interaction between the polyanionic editing RNAs and the cationic aminoglycosides we analyzed the thermal stability of the individual RNA helices at increasing Na^+^-ion concentrations (5–250mM) in the absence and presence of neomycin B. According to polyelectrolyte theory the melting temperature (T_m_) varies logarithmically with the salt concentration and the slope of the linear relationship is proportional to the difference in bound counter ions (Δn) in the folded and unfolded RNA. Fig. 4A–D summarize the results. In the absence of neomycin B all four T_m_ = f(log c_Na+_) plots are characterized by positive slopes (∂T_m_/∂logc_Na+_) indicating a release of counter ions upon RNA melting. Conversely, in the presence of 13μM neomycin B (13-fold over K_d_) the slopes are negative demonstrating an uptake of Na^+^-ions during melting. This implies that the temperature-induced strand separation of the individual RNA helices causes the bound aminoglycoside molecules to dissociate thereby freeing negative charges, which are neutralized by an uptake of Na^+^-ions. The calculated Δn-values vary from 1.8 to 2.6 in the absence of neomycin B and from-1.1 to-1.7 for the neomycin B-bound RNAs (Fig. 4E). Depending on the individual helices this indicates a release of 1 to 2 Na^+^-ions upon RNA melting and equally an uptake of 1 to 2 Na^+^-ions during melting of the neomycin B-complexed RNAs. The net difference between naked and neomycin B-complexed RNA helices calculates to ~3–4, which implies that in every helical element of the two editing RNAs 3–4 bound Na^+^-ions are replaced by 3–4 NH_3_^+^-groups of the aminoglycoside in order to facilitate the RNA/neomycin B interaction.

### Neomycin B binding does not alter the overall structure of the pre-mRNA/gRNA hybrid RNAs

**Fig 5:**
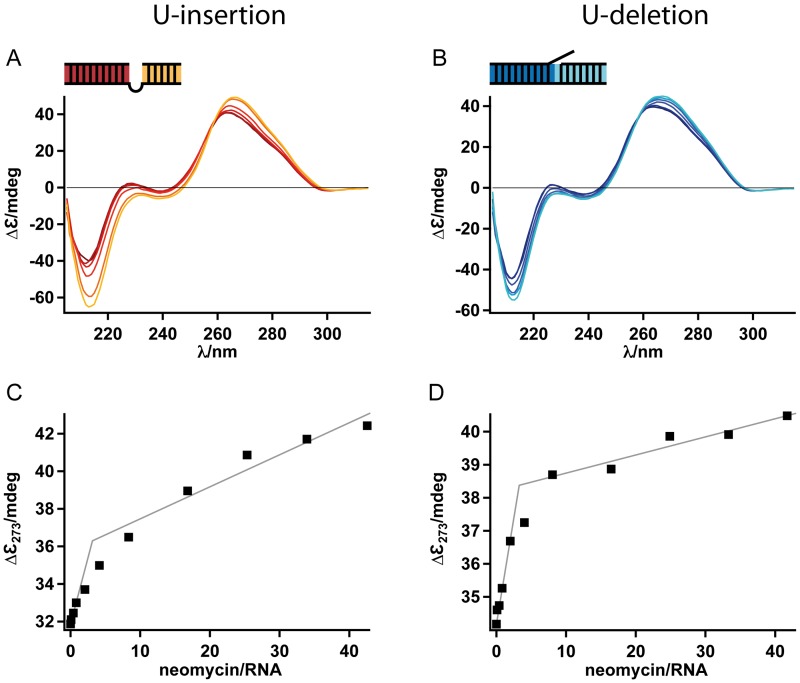
CD-spectra of RNA editing substrate RNAs at a concentration of 12μM. (A) U-insertion gRNA/pre-mRNA hybrid RNA (red trace). (B) U-deletion gRNA/pre-mRNA hybrid RNA (dark blue trace). Both spectra show typical A-form characteristics. Adding increasing concentrations of neomycin B (1.3μM-0.5mM) yields the spectra shown in orange to yellow (U-insertion) and light blue to cyan (U-deletion). (C/D) Plotting the spectral changes at 273nm as a function of the molar neomycinB/RNA ratio results in 3.1 (U-insertion) and 3.3 (U-deletion) neomycin binding sites per editing RNA.

To analyze whether the aminosugar-induced thermodynamic stabilization of editing substrate RNAs is a result of structural changes in the gRNA/pre-mRNA hybrid molecules we performed a conformational analysis using circular dichroism (CD) spectroscopy. Representative spectra are shown in Fig. 5A/B. As expected, both RNAs show the characteristics of A-form RNA [39]: a dominant negative signal at 212nm, a strong positive band at 265nm and a weak negative signal at roughly 300nm (with a crossover point at 224–234nm). The addition of neomycin B at molar ratios up to 40/1 alters both spectra in a concentration-dependent fashion although only moderately. Changes in the ellipticity indicate an increase in stacking [40] in line with the formation of a more compact structure of the two RNAs around the ligand binding sites [41]. However, the overall A-form geometry of the two RNAs is not altered upon binding of the aminosugar. Plotting the change in ellipticity (ΔƐ) at 273nm as a function of the molar neomycin B/RNA ratio yields in both cases saturation-type titration curves from which, in agreement with the above-described ITC- and UV-melting data, 3.1 (U-insertion) and 3.3 (U-deletion) binding sites were calculated (Fig. 5C/D).

### Modeling/docking of the neomycin B/editing RNA complexes

**Fig 6:**
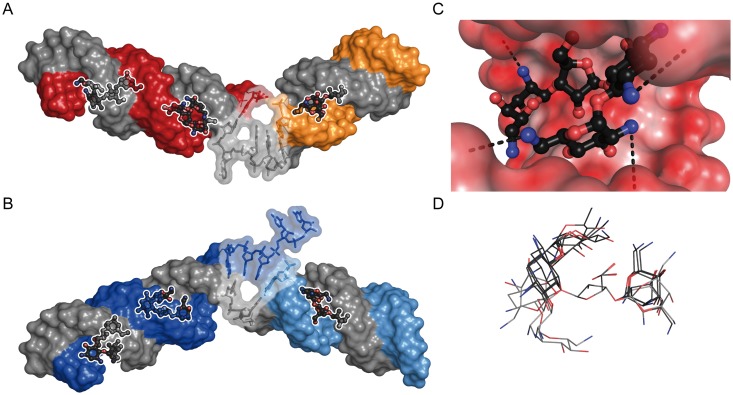
Docking-derived molecular models of neomycin B-complexed U-insertion (A) and U-deletion (B) gRNA/pre-mRNA hybrid RNAs. gRNA molecules are shown in grey; pre-mRNAs in red/yellow (U-insertion) and dark blue/light blue (U-deletion). Neomycin B is shown in a ball and stick representation (nitrogen-blue, oxygen-red, carbon-grey). Three neomycin B molecules are bound into the major groove of the individual RNA helices in line with the results from the ITC-, CD- and UV-melting experiments. The U-insertion gRNA/pre-mRNA hybrid RNA is bent by an angle of 134°, the U-deletion RNA by 138°. Nucleotides at the editing sites are depicted in a transparent envelop. (C) Calculated electrostatic surface potentials (red: negative; white: neutral; blue: positive) of a neomycin B binding site within the major groove of the U-deletion editing substrate. Possible ionic contacts are shown as dashed lines. (D) Conformational superposition of all 6 neomycin B molecules aligned to the ribose moiety of the molecule.

In order to gain a structural understanding of the aminoglycosid-mediated inhibition of editing we decided to model the two gRNA/pre-mRNA hybrid RNAs based on the above-described experimental data and to predict possible neomycin B binding sites by ligand docking. The two RNAs were interactively modeled with the help of ERNA3D^©^ 2.0 using standard A-form geometries for the helical elements and maximal stacking of the ss-nucleotides at the editing junction [42]. Neomycin B was docked into the two RNAs by blind docking using Autodock Vina 1.1.2 [35,43,44]. Neomycin B was predicted to bind into the major groove of the individual helices in both RNAs with in each case 9 possible conformations of very similar binding energies (variations between-38 and-41kJ/mol) and orientations. The pairwise root-mean-square deviation (RMSD) between the different conformations was maximally 3.3Å and minimally 0.5Å. Each neomycin B molecule covers a length of about 6bp, which allows the integration of 2 neo molecules into the larger helical elements in both RNAs and of 1 neomycin B molecule into the shorter stems in agreement with the calculated neomycin B/RNA stoichiometry of 3:1 (Fig. 6A/B). All predicted neomycin B conformations show 3–4 ionic contacts to the RNA backbone with an average distance of 3.7Å of an amino-nitrogen of the ligand to an oxygen atom of a phosphate group (Fig. 6C). Neomycin B binding does not distort the helical geometries of the two RNAs and a superposition of the six different neo conformations within their binding pockets is shown in Fig. 6D.

## Discussion

The RNA editing reaction within the mitochondria of African trypanosomes represents an ideal drug target for two reasons: First, editing is a required biochemical pathway for the survival of the parasite and second, the reaction cycle involves a multi-enzyme machinery (the editosome) and a class of small, non-coding RNAs (gRNAs) that only exist in the parasite. Although some editosome-interacting compounds have been identified [15], no inhibitor targeting the substrate gRNA/pre-mRNA hybrid RNAs of the processing reaction is known to date.

Here we demonstrate that the aminosugars neamin, neomycin B, tobramycin and sisomycin inhibit RNA editing at micromolar concentrations. These values are in a similar range to the inhibitory concentrations for other RNA-driven biochemical processes such as the neomycin B-induced inhibition of the hammerhead ribozyme [45], the inhibition of the human hepatitis delta virus self-cleavage RNA [46] or RNase P RNA [47]. The two editing sub-reactions (U-insertion/U-deletion) are repressed with similar qualitative and quantitative characteristics, which is in line with the fact that the two reactions are catalyzed out of a single RNA substrate binding site [13]. In agreement with observations in other RNA-driven systems we demonstrate that the inhibitory effect is a direct consequence of RNA binding. The aminoglycoside-gRNA/pre-mRNA complexes have micromolar affinities and are characterized by a stoichiometry of 3 aminosugars per gRNA/pre-mRNA hybrid. The RNA binding sites localize to the major groove of the two helical domains of the hybrid RNAs with a ratio of about 1 aminoglycoside/10bp. This agrees with published data in other systems [24,26,48]. The binding reaction represents a quasi “shape readout” of the helical structure of the two gRNA/pre-mRNA hybrid RNAs, which is consistent with NMR and X-ray studies that have shown that the conformational specificity of aminosugars is a result of a set of favorable interactions deep in the major groove of A-form duplex structures [49–51,41]. Importantly, the binding reaction does not induce a gross structural perturbation of the A-form geometry of the two RNA helices, instead it results in a thermodynamic stabilization in the range of-7 to-23kJ/mol. This could be the result of a charge neutralization between the cationic aminoglycosides and the polyanionic RNAs [52,53], especially since at least three NH_3_^+^ groups contribute to a single neomycin B-gRNA/pre-mRNA interaction. Within the context of the editing reaction this suggests that the editosome/substrate RNA interaction relies on a defined number of negative charges, which when neutralized cause the processing reaction to stall. This is further supported by the fact that *in vitro* RNA editing is performed at low monovalent cation concentrations (≤30mM) because the reaction is known to be sensitve to high salt conditions.

Furthermore, the data suggest that the processing reaction requires a defined structural flexibility of the gRNA/pre-mRNA hybrid RNAs. Aminosugar binding induces a stabilization or “stiffening” of the substrate RNAs, which leads to inhibition. This scenario is supported by the observation that editosomes are known to tolerate dynamic RNA rearrangements at editing sites simply because the primary sequence of the pre- and partially edited mRNAs is constantly changed as a result of the numerous U-insertions and deletions. An inability to re-organize the structure of the gRNA/pre-mRNA hybrid molecules might therefore stall the reaction. This is supported by the fact that editosomes have been shown to execute RNA chaperone activity to resolve folded RNA motifs [13] in addition to the involvement of RNA helicases [54–56]. Related phenomena have been described in the case of tobramycin, which when bound to tRNA^Asp^, locks the RNA in a non-functional conformation [57] and the kissing loop-mediated dimerization of HIV-1 RNA, which is stabilized by the binding of aminoglycosides thereby blocking the kissing loop to duplex conversion [58].

Importantly, the inhibitory effect is not a result of a direct structural interference of the aminosugar molecules at the editing site. This is supported by our CD and modeling/docking data. Instead, it’s a consequence of multiple binding events distal to the RNA editing site, which is transduced to the catalytic domain. A likely canditate for this mediator function might be the water structure of the substrate gRNA/pre-mRNA hybrid RNAs. Electrostatically confined water molecules in the 1^st^-hydration shell of RNAs have been shown to add to the plasticity of RNA/ligand interactions [59,60,23]. Therefore, we suggest that aminoglycoside-induced distal changes of the RNA hydration shell transduce to the editing site to stall the processing reaction indirectly. This hypothesis is supported by the fact that *in vitro* RNA editing is inhibited at crowded solution conditions [16]. Synthetic crowding agents such as polyethylene glycol (PEG) are known to affect the hydration state and as a consequence the functionality of nucleic acid molecules [61,62].

The described results can be used to define a set of criteria to search for improved RNA-interacting editing inhibitors for instance by structure-guided approaches as in the case of HIV-1 RNA [63] or by virtually screening small molecules to target the dynamic structural landscape of the substrate gRNA/pre-mRNA molecules [64]. The compounds should accommodate scaffolds to allow for a “shape readout” of RNA helices. They should be able to compensate the local negative charges of the substrate RNAs and induce some form of rigidity into the gRNA/pre-mRNA hybrid RNAs. Whether that is possible with compounds that abandon the aminoglycoside chemistry in favor of other RNA-interacting chemical scaffolds remains to be tested [23].

## Supporting Information

S1 Fig Structures of neamin and all tested 4,5–2-desoxystreptamines (2-DOS), 4,6–2-desoxystreptamines, aminocyclitoles and macrolides. Roman numbers annotate the different ring structures, arabian numbers label individual C-atoms. Ring II represents the core 2-DOS scaffold. Grey lines are used to delimit the individual aminoglycosides. The various NH_2_- or OH-substituents that distinguish the different aminosugars are listed next to their names. Sisomicin and geneticin, both 4,6–2-DOS-linked aminoglycosides, share a common scaffold consisting of rings II and III.
(TIF)

S2 Fig Primary sequences of *in vitro* RNA editing substrate RNAs. Top: pre-cleaved U-insertion gRNA/pre-mRNA hybrid RNA. Bottom: pre-cleaved U-deletion gRNA/pre-mRNA. gRNA-“guiding” nucleotides are in grey. U-nucleotides to be deleted are in black.
(TIF)

S3 Fig Dose response curves for the inhibition of U-insertion (A) and U-deletion RNA editing (B) by neamin. Radioactively labelled (5’-^32^P) gRNA/pre-mRNA substrate RNAs were incubated with 20S editosomes in the presence of increasing concentrations of neamin (1.6μM-1.7mM). The formation of edited products is plotted as a function of the neamin concentration to derive half-maximal inhibitory concentrations (IC_50_). Errors are standard deviations (s.d.).
(TIF)

S4 Fig Neomycin B inhibition of the endonucleotic cleavage step of the editing reaction cycle. The depicted radioactively labelled pre-mRNA/gRNA substrate RNA was incubated with 20S editosomes in the presence of increasing concentrations of neomycin B (0, 1, 3, 10, 30, 100, 1000μM; left to right). Endonucleolytic cleavage at the editing site (arrow) generates a 5’-mRNA cleavage fragment that was electrophoretically separated and densitometrically quantified (bar graph). M: mock treated sample. *: position of the radioactive label (^32^P).
(TIF)

S5 Fig Formation of neomycin B/editing RNA complexes of different stoichiometry using a sequential saturation binding site model. The graph shows the result of a numeric simulation of the concentration-dependent probability of forming neo/RNA complexes with one (yellow), two (orange) or three (red) neomycin B molecules per substrate RNA. Free RNA is shown in black. Half-maximal occupancy of all three binding sites is achieved at ~12μM (dashed grey line) in agreement with the derived IC_50_-value for the inhibition of the editing reaction. Fifty percent of the free RNA are complexed at ~0.8μM (dahed grey line), which agrees with the macroscopic K_d_ of the neo/RNA interaction.
(TIF)

S6 Fig Neomycin B titration of 20S editosomes. Surface plasmon resonance-based time trace of surface-immobilized 20S editosomes (20S ed) incubated with increasing concentrations of neomycin B (1xK_d_, 10xK_d_, 100xK_d_). Measurements were performed as in [65]. Even at neomycin B concentrations 10-fold above the determined K_d_ of the RNA/neomycin B complex no binding was observed. Only at a ≥100-fold excess a weak interaction is visible.
(TIF)

S7 Fig Neomycin B titration of 20S editosome/RNA complexes. Surface plasmon resonance-based time trace of surface-immobilized 20S editosomes (20S ed) complexed with unedited *T*. *brucei* apocytochrome b (Cyb) mRNA (blue trace) and further incubated with neomycin B (10xK_d_, 100xK_d_, 250xK_d_) (red traces). Measurements were performed as in [65]. Even at the highest neomycin B concentration no disruption of the RNA/editosome complex can be detected (dashed line).
(TIF)

S1 Table Sequences and selected properties of RNA oligonucleotides used in this study. 3’-end amino modifications in two of the oligoribonucleotides were introduced to prevent self ligation.
(TIF)
