# Supplementary figures and images for: Charge Reduction and Thermodynamic Stabilization of Substrate RNAs Inhibit RNA Editing
Source: PLoS One. 2015 Mar 5;10(3):e0118940. doi: 10.1371/journal.pone.0118940 (PMC4350841; doi:10.1371/journal.pone.0118940)

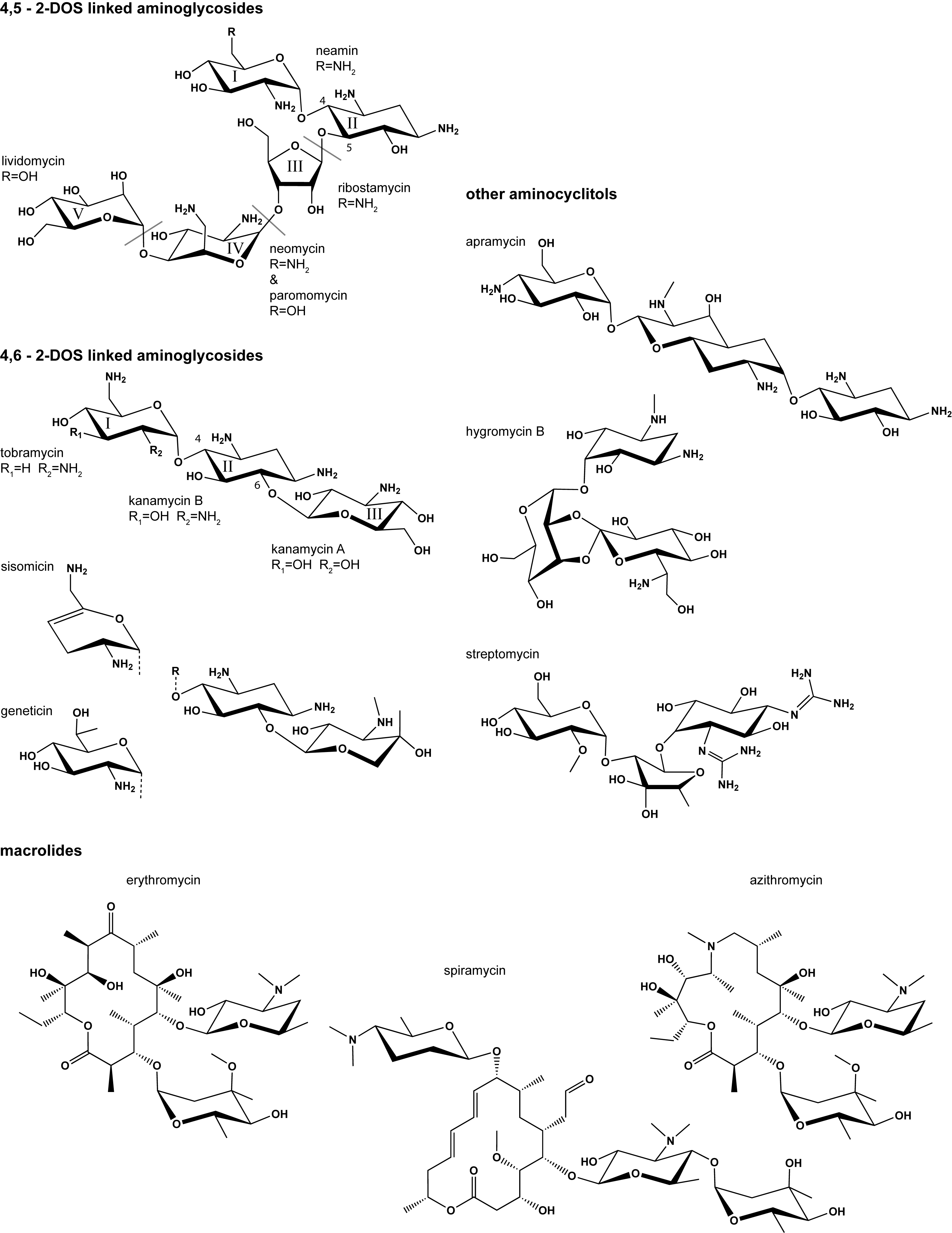

Supplement: S1 Fig — Roman numbers annotate the different ring structures, arabian numbers label individual C-atoms. Ring II represents the core 2-DOS scaffold. Grey lines are used to delimit the individual aminoglycosides. The various NH2- or OH-substituents that distinguish the different aminosugars are listed next to their names. Sisomicin and geneticin, both 4,6–2-DOS-linked aminoglycosides, share a common scaffold consisting of rings II and III. (TIF) [file pone.0118940.s001.tif]

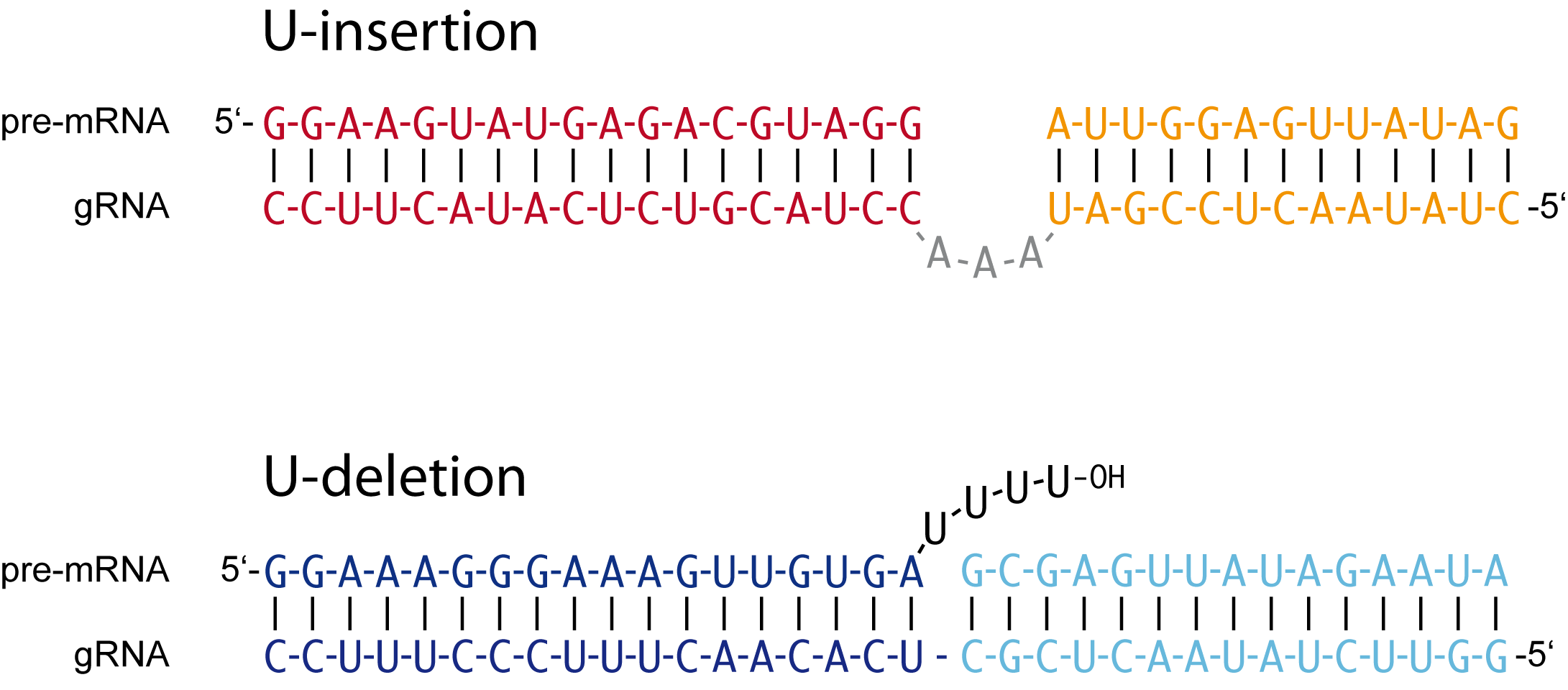

Supplement: S2 Fig — Top: pre-cleaved U-insertion gRNA/pre-mRNA hybrid RNA. Bottom: pre-cleaved U-deletion gRNA/pre-mRNA. gRNA-“guiding” nucleotides are in grey. U-nucleotides to be deleted are in black. (TIF) [file pone.0118940.s002.tif]

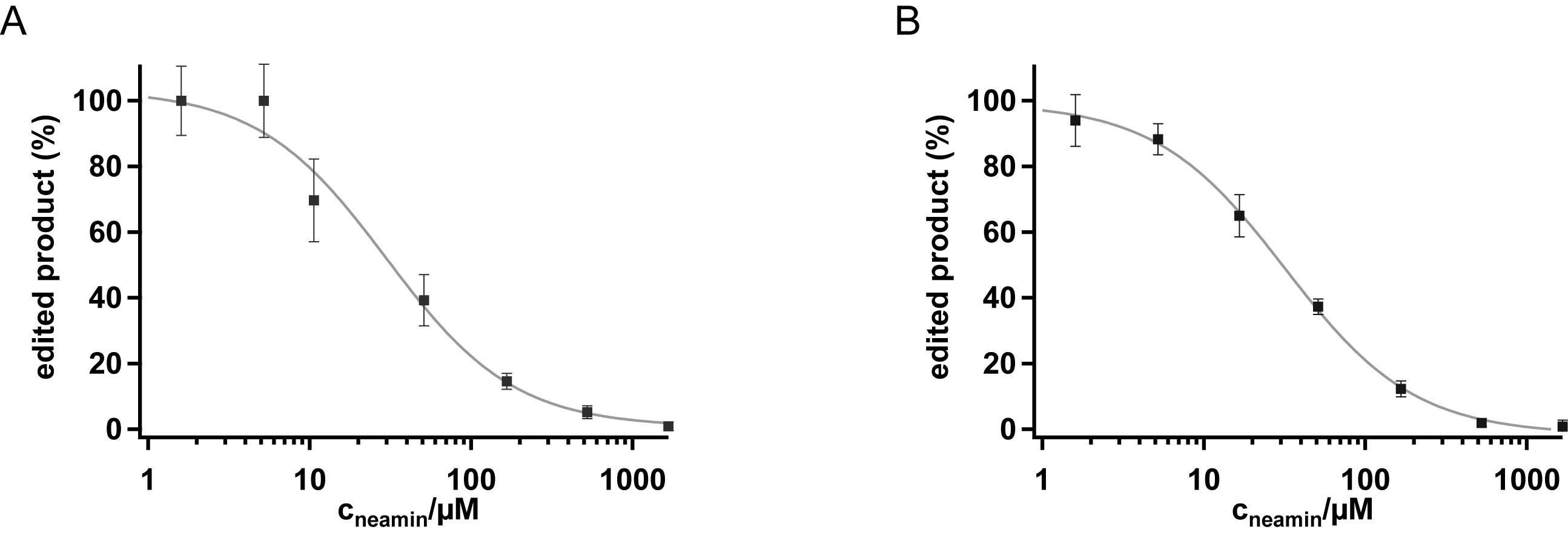

Supplement: S3 Fig — Radioactively labelled (5’-32P) gRNA/pre-mRNA substrate RNAs were incubated with 20S editosomes in the presence of increasing concentrations of neamin (1.6μM-1.7mM). The formation of edited products is plotted as a function of the neamin concentration to derive half-maximal inhibitory concentrations (IC50). Errors are standard deviations (s.d.). (TIF) [file pone.0118940.s003.tif]

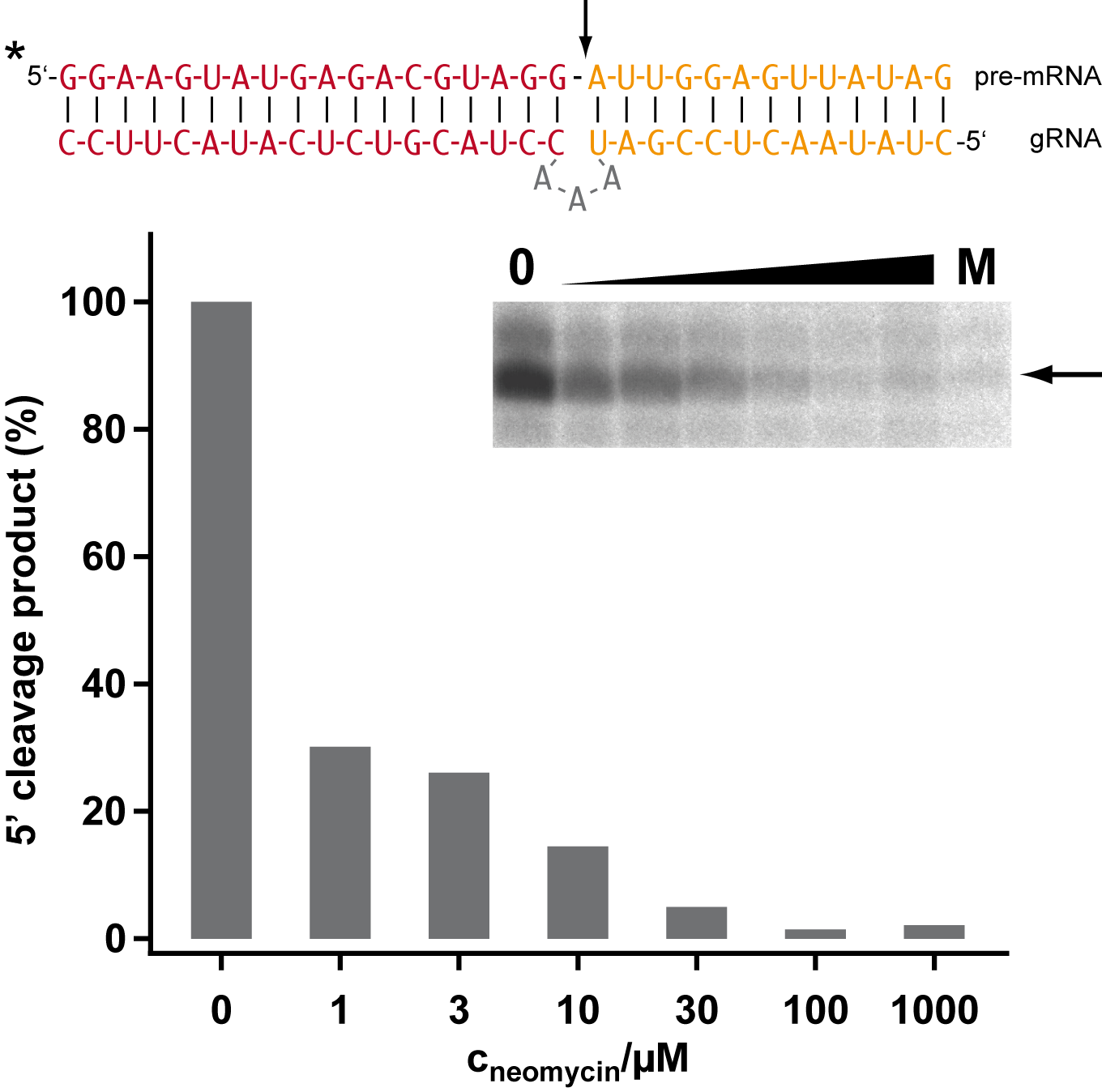

Supplement: S4 Fig — The depicted radioactively labelled pre-mRNA/gRNA substrate RNA was incubated with 20S editosomes in the presence of increasing concentrations of neomycin B (0, 1, 3, 10, 30, 100, 1000μM; left to right). Endonucleolytic cleavage at the editing site (arrow) generates a 5’-mRNA cleavage fragment that was electrophoretically separated and densitometrically quantified (bar graph). M: mock treated sample. *: position of the radioactive label (32P). (TIF) [file pone.0118940.s004.tif]

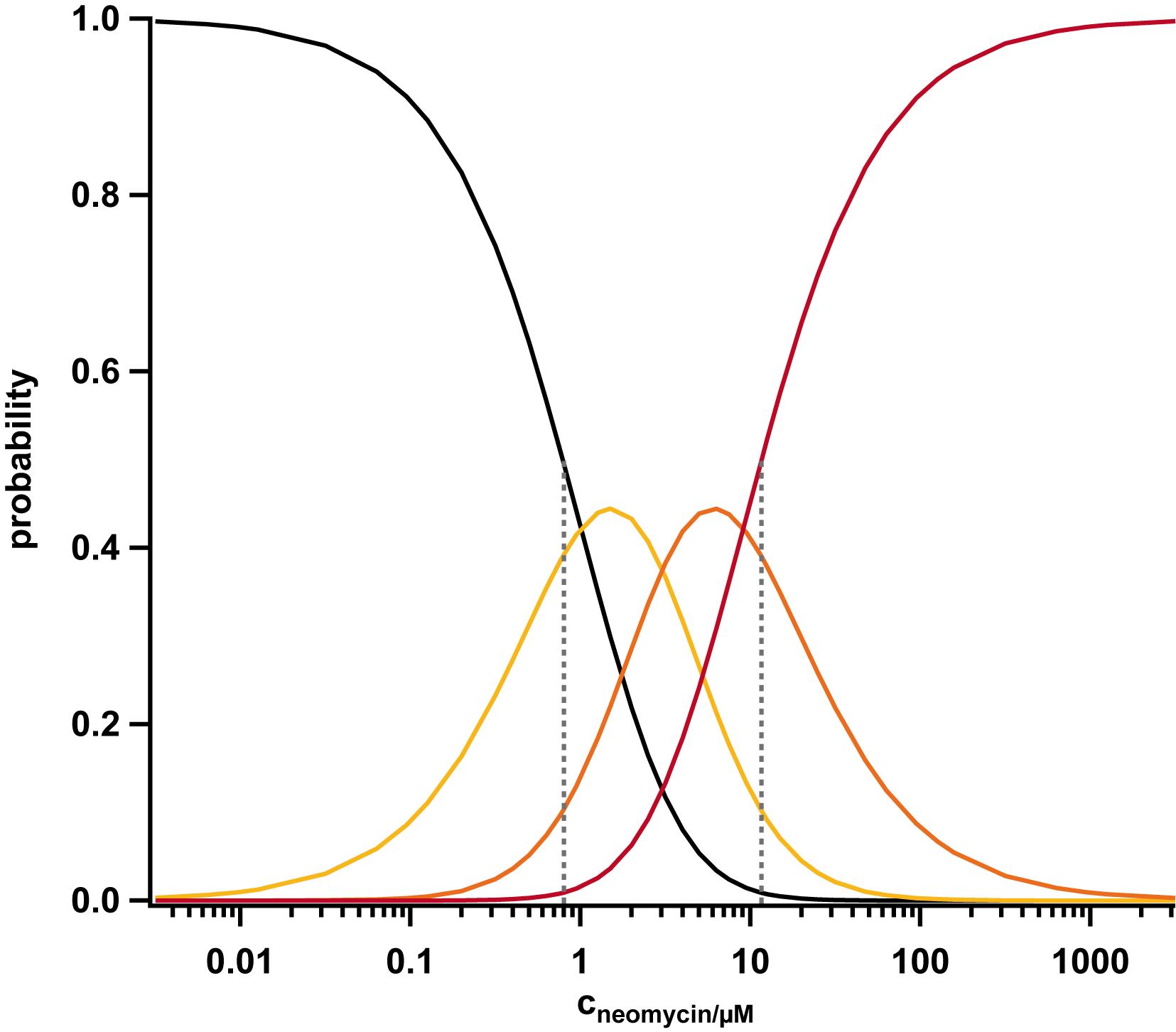

Supplement: S5 Fig — The graph shows the result of a numeric simulation of the concentration-dependent probability of forming neo/RNA complexes with one (yellow), two (orange) or three (red) neomycin B molecules per substrate RNA. Free RNA is shown in black. Half-maximal occupancy of all three binding sites is achieved at ~12μM (dashed grey line) in agreement with the derived IC50-value for the inhibition of the editing reaction. Fifty percent of the free RNA are complexed at ~0.8μM (dahed grey line), which agrees with the macroscopic Kd of the neo/RNA interaction. (TIF) [file pone.0118940.s005.tif]

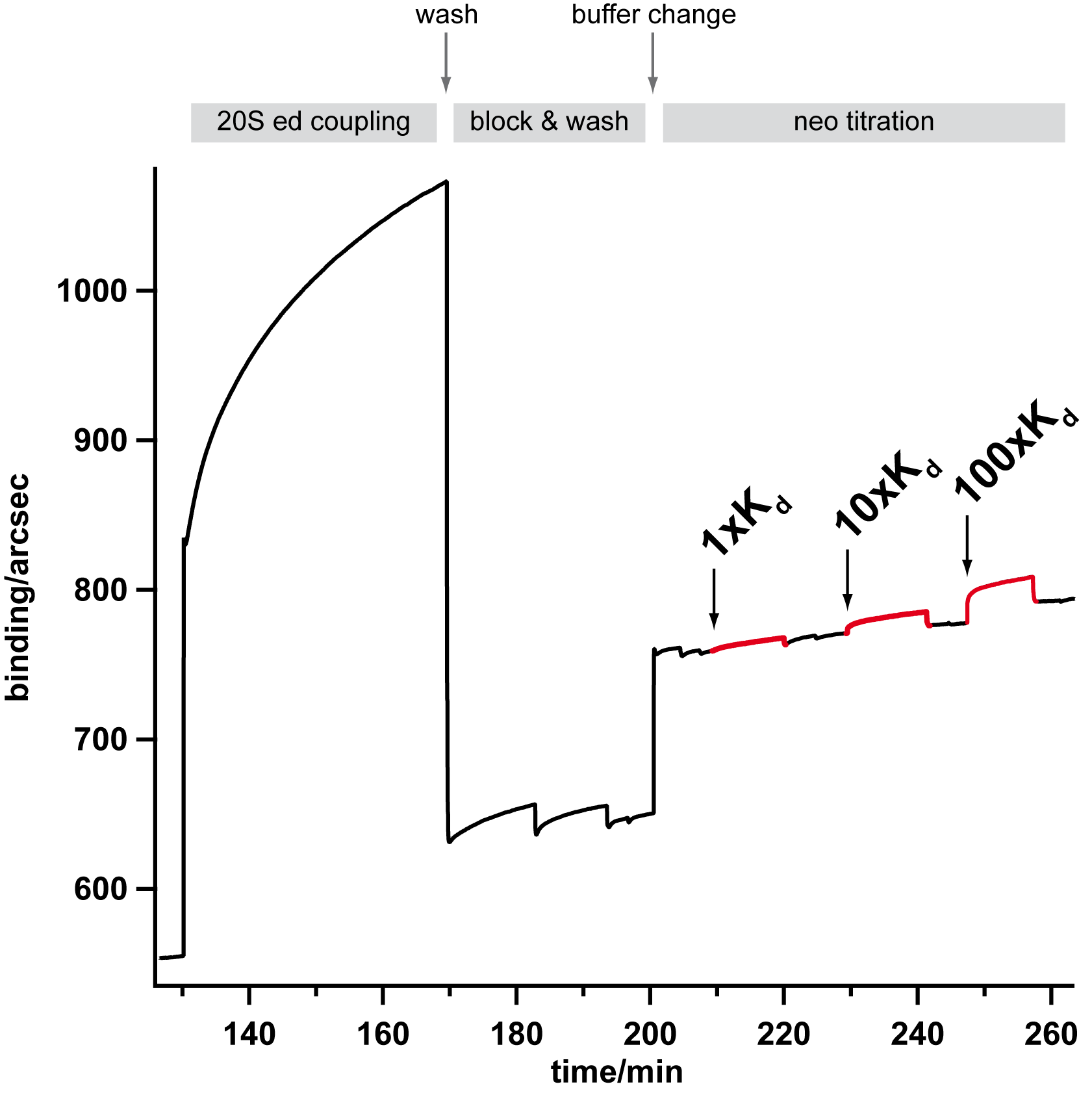

Supplement: S6 Fig — Surface plasmon resonance-based time trace of surface-immobilized 20S editosomes (20S ed) incubated with increasing concentrations of neomycin B (1xKd, 10xKd, 100xKd). Measurements were performed as in [65]. Even at neomycin B concentrations 10-fold above the determined Kd of the RNA/neomycin B complex no binding was observed. Only at a ≥100-fold excess a weak interaction is visible. (TIF) [file pone.0118940.s006.tif]

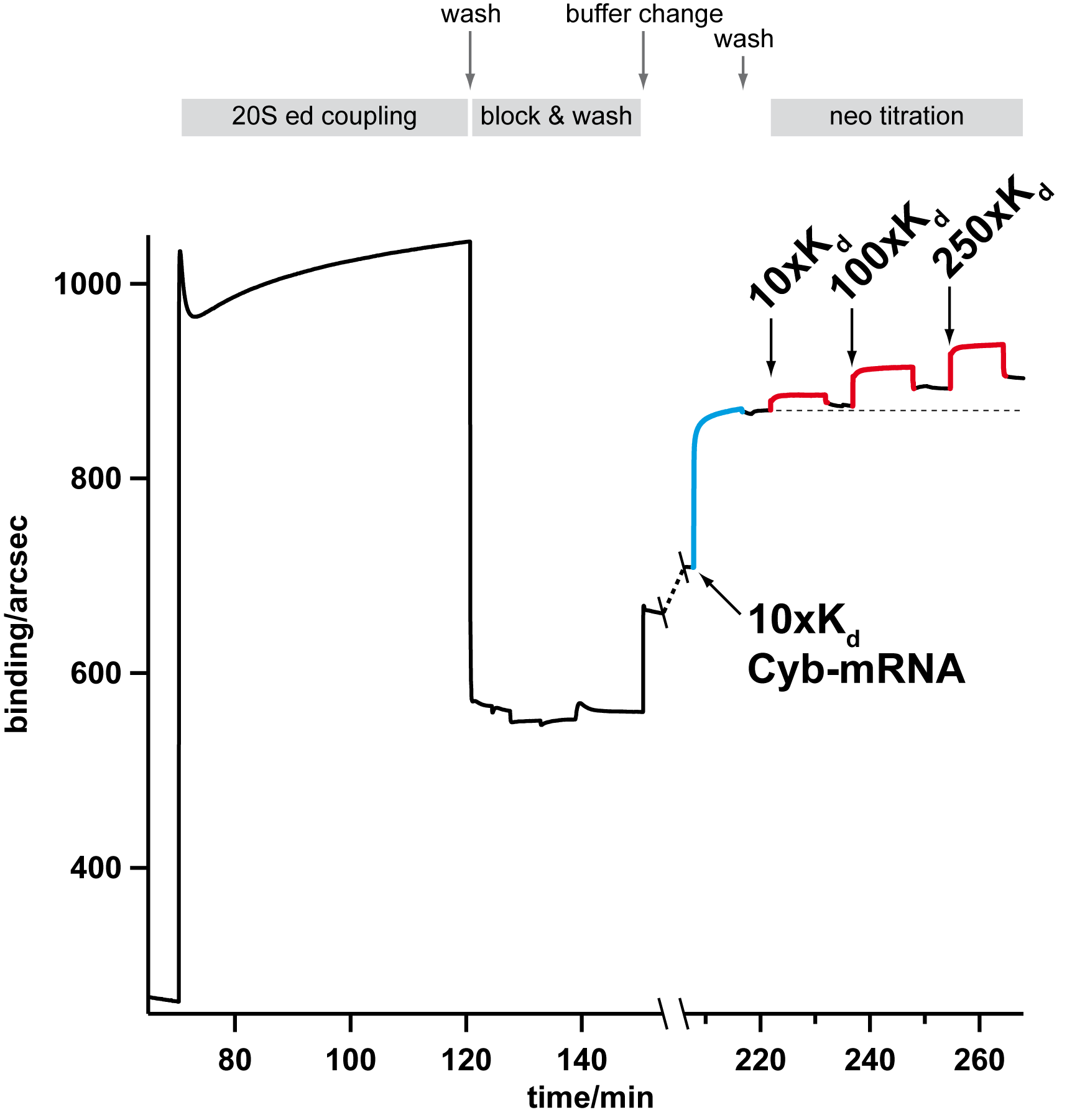

Supplement: S7 Fig — Surface plasmon resonance-based time trace of surface-immobilized 20S editosomes (20S ed) complexed with unedited T. brucei apocytochrome b (Cyb) mRNA (blue trace) and further incubated with neomycin B (10xKd, 100xKd, 250xKd) (red traces). Measurements were performed as in [65]. Even at the highest neomycin B concentration no disruption of the RNA/editosome complex can be detected (dashed line). (TIF) [file pone.0118940.s007.tif]

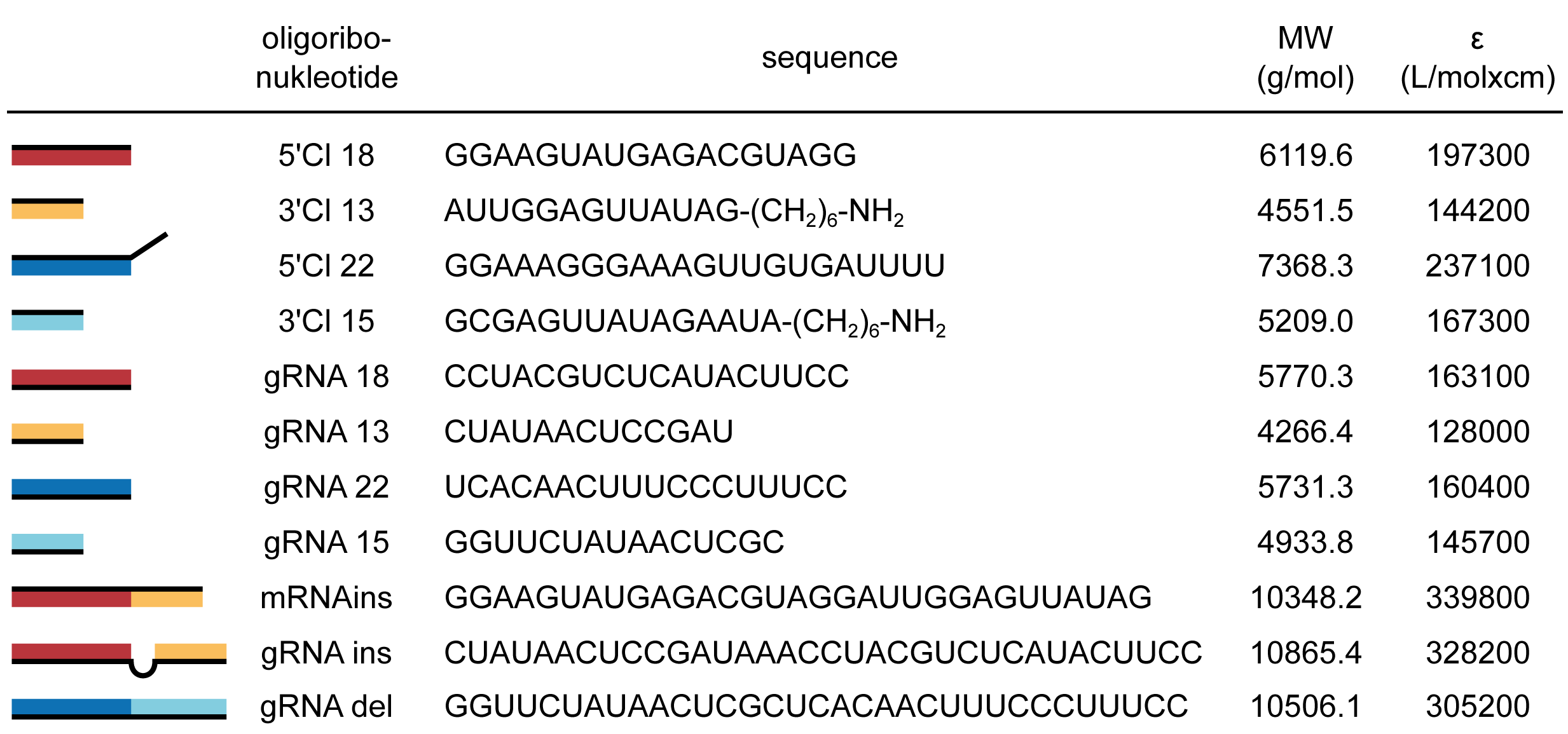

Supplement: S1 Table — 3’-end amino modifications in two of the oligoribonucleotides were introduced to prevent self ligation. (TIF) [file pone.0118940.s008.tif]
